# Acceptability and safety of thermal ablation for the treatment of precancerous cervical lesions in Honduras

**DOI:** 10.1111/tmi.13315

**Published:** 2019-11-21

**Authors:** Manuel Sandoval, Rose Slavkovsky, Pooja Bansil, Jose Jeronimo, Jeanette Lim, Jacqueline Figueroa, Silvia de Sanjose

**Affiliations:** ^1^ Asociación Hondureña de Planificación de Familia Tegucigalpa Honduras; ^2^ PATH Seattle WA USA; ^3^ Consulting Damascus MD USA; ^4^ Secretaría de Salud de Honduras Tegucigalpa Honduras

**Keywords:** pain, safety, thermal ablation, cervical cancer, human papillomavirus, Honduras, douleur, sécurité, ablation thermique, cancer cervical, VPH, Honduras

## Abstract

**Objective:**

To evaluate the acceptability and safety of thermal ablation (TA) for the treatment of precancerous cervical lesions in women in Honduras.

**Methods:**

Human papillomavirus (HPV) and visual inspection with acetic acid (VIA) screen‐positive eligible women received TA. After treatment, women rated the level of pain experienced during treatment using the Wong‐Baker FACES® pain‐rating scale from 0 to 10. Short‐term safety outcomes that could require medical attention were assessed one month after treatment.

**Results:**

A total of 319 women received TA treatment. The average pain rating was 2.5 (95% CI: 2.3–2.8), and 85% rated their pain levels as less than 6. No significant differences in low (below 6) or high (6 and above) pain were found by age or number of biopsies performed, but there was a significant difference by the number of TA applications (*P* < 0.01). When asked if they would recommend this treatment, all women said they would. At the one‐month follow‐up visit, the most common reported discomforts were bleeding (10%) and cramping (8.4%); 11 women reported severe lower abdominal pain, and none required medical attention.

**Conclusions:**

TA is safe and acceptable to patients as a treatment option for precancerous cervical lesions in low‐resource settings.

## Abstract (fr)

**Objectif:**

Evaluer l'acceptabilité et la sécurité de l'ablation thermique (AT) pour le traitement des lésions cervicales précancéreuses chez les femmes au Honduras.

**Méthodes:**

Les femmes éligibles, portant le virus du papillome humain (VPH) et avec une inspection visuelle positive au test de dépistage à l'acide acétique (IVA) ont reçu une AT. Après le traitement, les femmes ont estimé le niveau de douleur ressenti au cours du traitement à l'aide de l’échelle d'évaluation de la douleur Wong‐Baker FACES® de 0 à 10. Les résultats de sécurité à court terme pouvant nécessiter une attention médicale ont été évalués un mois après le traitement.

**Résultats:**

319 femmes ont reçu un traitement d’AT. L’estimation moyenne de la douleur était de 2,5 (IC95%: 2,3–2,8) et 85% estimaient que leur niveau de douleur était inférieur à 6. Aucune différence significative dans la douleur faible (moins de 6) ou élevée (6 et plus) n’a été constatée en fonction de l’âge ou du nombre de biopsies réalisées, mais il y avait une différence significative selon le nombre d'applications d’AT (*P* < 0,01). Lorsqu'on leur a demandé si elles recommanderaient ce traitement, toutes les femmes ont répondu qu'elles le feraient. Lors de la visite de suivi à un mois, les malaises les plus fréquents signalés étaient les saignements (10%) et les crampes (8,4%). Onze femmes ont rapporté des douleurs sévères dans le bas de l'abdomen et aucune n'a nécessité de soins médicaux.

**Conclusions:**

L'AT est sûre et acceptable pour les patientes comme option de traitement pour les lésions cervicales précancéreuses dans les régions à faibles ressources.

## Introduction

Cervical cancer is the second leading cause of cancer deaths in women in low and low–middle income countries 1. In Honduras, the crude incidence rate of cervical cancer is 17.0 cases per 100 000 women, with approximately 804 new cases and 480 deaths reported annually 2. Cervical cancer is largely preventable through human papillomavirus (HPV) vaccination and routine cervical screening and treatment of precancerous cervical lesions 3, 4. Honduras recently incorporated HPV testing into its national cervical cancer screening programmes, given the availability of less costly HPV DNA tests 5.

However, improvements in treatment technologies, a key component of effective cervical cancer screening programmes, have lagged behind advances in screening. For low‐resource settings, WHO recommends cryotherapy 6, an ablative procedure wherein visible precancerous cervical lesions are destroyed with probes cooled to freezing temperatures by compressed refrigerant gas. While cryotherapy is relatively easy to perform, has few side effects (pain and vaginal discharge) and does not have adverse effects on fertility 7, it has several limitations that inhibit wide implementation in public health systems in developing countries. The main limitation of cryotherapy is the need for refrigerant gas, which is expensive and challenging to procure, and the large, heavy storage tanks are difficult and costly to transport, which leads to unreliable supply 8, 9, 10. Additionally, the probes used to freeze lesions can malfunction. As a result, when cryotherapy cannot be performed due to non‐functional medical equipment or lack of critical supplies such as the gas, women may not receive timely treatment, or any treatment at all. Thus, alternative non‐gas treatment technologies such as thermal ablation may offer a more feasible treatment option in low‐resource settings 11. Research has shown that the rates of lesion disappearance with thermal ablation are as good or better than those achieved by conventional cryotherapy 12, 13, 14, 15 and cure rates have been reported to be >80% in observational studies 8, 9, 16, 17. Further, the procedure is reported to be safe, with few adverse events; it is quick 18, preserves fertility 9 and is well tolerated by women 8, 18, 19. However, evidence of client acceptability in low‐resource settings is still limited.

The objective of this study was to assess the acceptability and safety of thermal ablation among pre‐menopausal women for the treatment of precancerous cervical lesions in public health facilities in Honduras as a standard of care.

## Methods

### Study area

This study took place in four government health facilities in the metropolitan region of Francisco Morazán in Honduras, which includes the capital city of Tegucigalpa and has a population of 352 784 women of reproductive age (15–49 years). This region is one of the areas where the government is conducting a cervical cancer prevention programme that uses HPV testing as the primary screening strategy.

### Ethical approval

Institutional review board approvals were obtained for both the original study protocol and the amendments from PATH’s Research Ethics Committee and the Comité de Ética en Investigación Biomédica (CEIB) of Universidad Nacional Autónoma de Honduras in Tegucigalpa. The study is registered on clinicaltrials.gov (Identifier: NCT03510273).

### Study recruitment and procedures

Study staff recruited women with a positive HPV screening test over a period of five months. Women were eligible to participate if they were as follows: aged 30–49 years, not pregnant, HPV and visual inspection with acetic acid (VIA) positive, and eligible for ablative treatment per the following WHO guidelines: VIA lesion covers <75% of the cervix, the lesion does not enter the endocervical canal, the entire lesion can be visualised and covered by the treatment probe 6, and there is no suspicion of invasive cancer. The study was restricted to pre‐menopausal women to guarantee that the transformation zone would be visible when performing VIA.

Of a total of 2049 HPV‐positive women in the catchment area, we were unable to reach 1063 women. Of the remaining 986 women, 11 were pregnant, 164 had already received treatment and 28 declined the study invitation to attend a clinic for follow‐up evaluations with VIA, per standard of care in Honduras. Among the women contacted, 783 (79.4%) completed the VIA evaluation. Of these, 326 (41.6%) had a positive VIA result and were invited to participate in the study. Six women declined to participate, and one woman was not eligible for the thermal ablation procedure. Women who declined participation received standard cryotherapy treatment and the ineligible woman was referred for appropriate care. Written informed consent was obtained from 319 eligible women to participate in the study.

The thermal ablation device used in this study was the Liger Medical Thermocoagulator (Liger Medical LLC, Lehi, UT), a handheld, battery‐operated instrument with a probe consisting of a shaft and heated tip. The removable probes have different types of tips that vary in size and shape; in this study, the 16‐ and 19‐millimetre (mm) flat tips as well as a 19‐mm nipple tip were used for different lesions, as appropriate.

None of the eligible and consenting women were pregnant; all underwent pelvic examinations and received VIA, whereby the cervix is examined one minute after application of 5% acetic acid, which causes precancerous cervical lesions to turn white. Women with abnormal/acetowhite VIA results, and whose lesions qualified for ablative treatment as determined by the aforementioned criteria, had one or more directed biopsies taken with Kevorkian forceps and then received thermal ablation treatment with the Liger device. During the informed consent process, study staff explained the series of procedures that women would undergo (VIA, biopsy and treatment with thermal ablation) and that women would be asked about how they felt during the treatment procedure specifically.

Biopsies were taken to diagnose the grade of the precancerous cervical lesions, which were categorised as cervical intraepithelial neoplasia (CIN) 1, 2 or 3, or as invasive cancer.

The Liger devices were set for a treatment cycle of 45 s at 100 °C, and the provider applied up to three treatment cycles to cover the whole transformation zone, as determined necessary. Analgesia (NSAIDS) was offered to women post‐treatment for pain management if necessary. All women received counselling on post‐treatment care and were scheduled for a one‐month follow‐up visit.

Immediately after the procedure, study nurses interviewed the women and asked them to report the level of pain experienced during the thermal ablation treatment using the Wong‐Baker FACES® pain rating scale 20. This validated scale consists of an illustrated series of six faces with a range of emotions from happy (0) to crying (10) to indicate level of pain. Scores are numbered from 0 to 10 in increments of two. After an initial period of data collection in the study, it was not clear if the pain women reported was associated with the ablation treatment or the biopsy procedure, and whether they received pain medication immediately following the treatment. Because of this uncertainty, and in order to accurately evaluate pain experienced due to thermal ablation, study procedures to solicit pain ratings were revised twice during the remaining study period.

In the first pain‐assessment time period (*n* = 55), post‐procedure pain medication was given routinely at some geographic sites, and at all sites, pain scores were solicited without adequate clarification that the woman should score pain experienced only during the thermal ablation treatment, and not during the biopsy. In the second pain‐assessment period (*n* = 174), across all sites, study staff did not routinely provide pain medication after the procedure, but they did continue to solicit pain scores without adequately clarifying that only pain experienced during the thermal ablation treatment should be reported. In the last pain‐assessment time period (*n* = 90), across all sites, study staff did not routinely provide pain medication after the procedure, and study staff clarified that only pain experienced during the thermal ablation treatment should be reported.

At the one‐month follow‐up visit, women received their biopsy results and underwent another pelvic examination to assess short‐term safety outcomes per WHO guidelines for ablative treatment. Safety was evaluated by asking women to report any of the following discomforts experienced during or immediately after the thermal ablation procedure: cramping, nausea, dizziness, faintness, flushing, bleeding or vaginal burns. They were also asked about problems experienced in the month after treatment, such as discharge, bleeding, pain while urinating, abdominal pain or fever. Based on their results, women were referred to additional treatment, as necessary.

### Statistical analysis

The distribution of self‐reported pain scores from the Wong‐Baker FACES® pain rating scale during the procedure was evaluated. Descriptive statistics of the following characteristics were calculated: geographic site, pain‐assessment time period (1, 2 or 3), age, number of biopsies, number of probe applications, type of probe used and biopsy results. In addition, these characteristics were compared among women with reported low pain scores (<6) and high pain scores (6+).

To investigate factors that might be associated with high pain scores, we sub‐categorised geographic sites (Crucitas, other), age (30–34 years, 35–39 years, and 40 years and older), number of treatment applications and number of biopsies (1 and ≥2). Poisson regression with robust variance was used to determine the prevalence of high pain scores (reported during procedures (prevalence ratios [PR]; 95% confidence intervals [CI]) compared with low pain. A multivariable model with categorised low or high pain score as the dependent variable was then applied, including all variables, to estimate adjusted PRs. Statistical analyses were conducted in Stata 13.1 (Statacorp, College Station, TX, USA).

## Results

All 319 (100%) women enrolled in the study underwent thermal ablation. Sociodemographic characterisation, distribution of clinical procedures performed and biopsy results are shown in Table 1. The majority of women were from Crucitas (63.0%), were evaluated during pain‐assessment time period 2 (54.6%), were aged between 30 and 34 years (37.3%), had one biopsy taken (79.9%) and received one thermal ablation application (59.3%). Over 50% of women had a normal biopsy and 24% had CIN2‐3. Two women were diagnosed with an invasive cancer and were referred for treatment.

**Table 1:** Sociodemographic characteristics, clinical procedures and outcomes among participants who received thermal ablation treatment (*N* = 319)

	Number	Per cent
Total	319	100
Site		
Carrizal	62	19.4
Crucitas	201	63.0
Los Pinos/San Benito	44	13.8
San Miguel	12	3.8
Pain assessment period*		
1	55	17.2
2	174	54.6
3	90	28.2
Age, years		
30–34	119	37.3
35–39	102	32.0
40–44	62	19.4
45–49	36	11.3
Number of biopsies		
1	255	79.9
2	58	18.2
3	6	1.9
Number of thermal ablation applications		
1	189	59.3
2	113	35.4
3	17	5.3
Liger Thermocoagulator probe tip used on individuals with 1 application (*N* = 189†)		
Nipple 19 mm tip	104	55.0
Flat 19 mm tip	85	45.0
Liger Thermocoagulator probe tip used on individuals with ≥ 2 applications (*N* = 130‡)		
Nipple 19 mm tip	143	51.6
Flat 19 mm tip	131	47.3
Flat 16 mm tip	3	1.1
Biopsy Results (*N* = 317§)		
Normal	160	50.5
CIN1	79	24.9
CIN2	40	12.6
CIN3	36	11.4
Cancer	2	0.6

CIN, cervical intraepithelial neoplasia.

* Pain scores were ascertained differently during the study period, as follows: Period 1: pain medication given routinely and no clarification of when pain scores were to be reported; Period 2: pain medication not given routinely and no clarification of when pain scores were to be reported; and Period 3: pain medication not given routinely and pain scores ascertained for thermal ablation treatment only.

† 189 study participants received only one thermal ablation application.

‡ 130 study participants had two or more thermal ablation applications.

§ Biopsy results for 2 study participants were not available: 1 participant was lost to follow‐up at Visit 2, and 1 participants’ biopsy was damaged during transportation.

**Figure 1:**
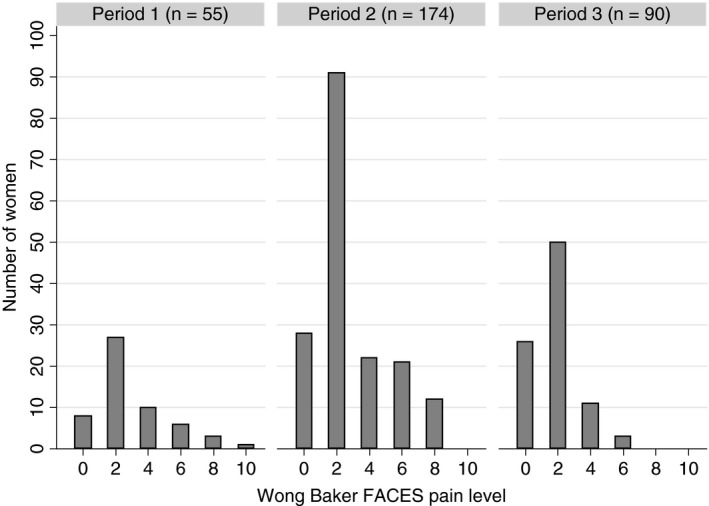
Level of pain using Wong‐Baker FACES® pain rating scale by pain assessment period among participants who received thermal ablation treatment. Pain scores were ascertained differently during the study period, as follows: Period 1: pain medication given routinely and no clarification of when pain scores were to be reported; Period 2: pain medication not given routinely and no clarification of when pain scores were to be reported; and Period 3: pain medication not given routinely, and pain scores ascertained for the thermal ablation treatment only.

Table 2 shows the distribution of reported pain scores on the Wong‐Baker FACES® pain rating scale from 0 to 10. The average pain rating was 2.5 (95% CI: 2.3–2.8), with 4.7% and 0.3% reporting pain as 8 and 10, respectively. Figure 1 shows the distribution of pain scores by pain‐assessment time period. Over all three periods, the majority of women (85.6%) reported their level of pain to be less than 6, while pain scores of 8 and 10 were only observed during the first and second time periods, during which it was not well clarified that pain was to be reported only for the thermal ablation procedure, and not for the biopsy. It was never necessary to stop the thermal ablation procedure by patient request because of pain.

**Table 2:** Pain scores using Wong‐Baker FACES® pain rating scale among participants who received thermal ablation treatment (*N* = 319)

Wong‐Baker FACES® levels of pain*	Overall *N* (%)	Pain assessment period† *N* (%)		
		1 = 55	2 = 164	3 = 90
0	62 (19.4)	8 (14.5)	18 (11.0)	26 (28.9)
2	168 (52.7)	27 (49.1)	91 (55.5)	50 (55.6)
4	43 (13.5)	10 (18.2)	22 (13.4)	11 (12.2)
6	30 (9.4)	6 (10.9)	21 (12.8)	3 (3.3)
8	15 (4.7)	3 (5.5)	12 (7.3)	0
10	1 (0.3)	1 (1.8)	0	0

* While the numbers for scoring pain range from zero to ten, the pain scale consists of just six faces showing different responses to pain experienced.

† Pain scores were ascertained differently during the study period, as follows: Period 1: pain medication given routinely and no clarification of when pain scores were to be reported; Period 2: pain medication not given routinely and no clarification of when pain scores were to be reported; and Period 3: pain medication not given routinely, and pain scores ascertained for the thermal ablation treatment only.

Across all sites, there was no statistically significant difference between women with low or high pain scores by age or number of biopsies (Table 3). There was a significant difference in level of pain reported by number of treatment applications, with more pain reported by those receiving ≥2 applications. Overall, there was no clear relationship between the type of probe tip used for treatment and women’s reported level of pain. Higher levels of pain were reported with the use of nipple probe tips compared with flat probe tips among women who received one application (*P* = 0.03), but this association did not hold true among women who received more than one application (*P* = 0.52).

**Table 3:** Sociodemographic characteristics, clinical procedures and outcomes, by self‐reported pain score*, among participants who received thermal ablation treatment (*N* = 319)

	Total *n* (%)	Low Pain (<6)* *n* (%)	High Pain (6+)* *n* (%)	*P* value‡
Total	319	273 (85.6)	46 (14.4)	
Site				
Carrizal	62	62 (100.0)	0 (0)	<0.01
Crucitas	201	161 (80.1)	40 (19.9)	
Los Pinos/San Benito	44	40 (90.9)	4 (9.1)	
San Miguel	12	10 (83.3)	2 (16.7)	
Pain assessment period†				
1	55	45 (81.8)	10 (18.2)	<0.01
2	174	141 (81.0)	33 (19.0)	
3	90	87 (96.7)	3 (3.3)	
Age, years				
30–34	119	98 (82.3)	21 (17.7)	0.33
35–39	102	86 (84.3)	16 (15.7)	
40–44	62	57 (91.9)	5 (8.1)	
45–49	36	32 (88.9)	4 (11.1)	
Number of biopsies				
1	255	222 (87.1)	33 (12.9)	0.15
2	58	47 (81.0)	11 (19.0)	
3	6	4 (66.7)	2 (33.3)	
Number of thermal ablation applications				
1	189	171 (90.5)	18 (9.5)	0.01
2	113	88 (77.9)	25 (22.1)	
3	17	14 (82.4)	3 (17.7)	
Liger Thermocoagulator probe tip used on individuals with 1 application (*N* = 189§)				
Nipple 19mm tip	104	90 (86.5)	14 (13.5)	0.03
Flat 19 mm tip	85	81 (95.3)	4 (4.7)	
Liger Thermocoagulator probe tip used on individuals with ≥ 2 applications (*N* = 130‖)				
Nipple 19mm tip	143	115 (80.4)	28 (19.6)	0.52
Flat 19 mm tip	131	101 (77.1)	30 (22.9)	
Flat 16mm tip	3	2 (66.7)	1 (33.3)	
Biopsy Results (*N* = 317¶)				
Normal	160	129 (80.6)	31 (19.4)	0.07
CIN1	79	75 (94.9)	4 (5.1)	
CIN2	40	34 (85.0)	6 (15.0)	
CIN3	36	31 (86.1)	5 (13.9)	
Cancer	2	2 (100.0)	0 (0)	

CIN, cervical intraepithelial neoplasia.

* Self‐reported pain scores from Wong‐Baker FACES® pain rating scale were categorised as low pain (<6) and high pain (>6).

^†^ Pain scores were ascertained differently during the study period, as follows: Period 1: pain medication given routinely and no clarification of when pain scores were to be reported; Period 2: pain medication not given routinely and no clarification of when pain scores were to be reported; and Period 3: pain medication not given routinely, and pain scores ascertained for the thermal ablation treatment only.

^‡^ Fisher exact* P* value comparing the distribution between low and high pain.

§ 189 study participants received only one thermal ablation application.

‖ 130 study participants had two or more thermal ablation applications.

¶ Biopsy results for 2 study participants were not available: 1 participant was lost to follow‐up at Visit 2, and 1 participants’ biopsy was damaged during transportation.

Table 4 describes the PR of high pain *vs.* low pain by factors that could be associated with pain scores. Adjusted results are presented by geographic site, pain‐assessment time period, age, number of biopsies and number of treatment applications. Women evaluated at Crucitas had significantly higher PR of high pain scores (PR: 5.62; 95% CI: 2.44–12.91) while women evaluated in the third pain‐assessment time period—when it was clarified that pain should be reported only for the thermal ablation procedure—had significantly lower PR (PR: 0.17; 95% CI: 0.04–0.63).

**Table 4:** Factors associated with high pain score among participants who received thermal ablation treatment (*N* = 319)

	Unadjusted* Prevalence Ratio (95% CI)	Adjusted† Prevalence Ratio (95% CI)
	*n* (%)	*n* (%)
Site		
Carrizal/Los Pinos/San Benito/ San Miguel	Ref	Ref
Crucitas	3.91 (1.71–8.96)	5.62 (2.44–12.91)
Pain assessment period‡		
1	Ref	Ref
2	1.04 (0.55–1.98)	1.22 (0.68–2.2)
3	0.18 (0.05–0.64)	0.17 (0.04–0.63)
Age, years		
30–34	Ref	Ref
35–39	0.89 (0.49–1.61)	1.00 (0.57–1.76)
40+	0.52 (0.25–1.09)	0.58 (0.29–1.16)
Number of biopsies		
1	Ref	Ref
≥ 2	1.57 (0.88–2.81)	0.69 (0.36–1.33)
Number of thermal ablation applications		
1	Ref	Ref
≥2	2.26 (1.31–3.91)	1.59 (0.85–2.98)

CI, confidence interval; Ref, Reference.

* Unadjusted prevalence ratio for the association between each factor and high pain score (*vs.* a low pain score).

^†^ Adjusted prevalence ratio for all the listed factors and high pain score (*vs.* a low pain score).

^‡^ Pain scores were ascertained differently during the study period, as follows: Period 1: pain medication given routinely and no clarification of when pain scores were to be reported; Period 2: pain medication not given routinely and no clarification of when pain scores were to be reported; and Period 3: pain medication not given routinely, and pain scores ascertained for the thermal ablation treatment only.

Table 5 describes the safety outcomes at the one‐month follow‐up visit after the treatment. Very few women experienced discomfort during or immediately after the procedure; the most common discomfort reported was bleeding/spotting (10%), followed by mild cramping (7.5%) and dizziness (5%). At one month after treatment, the most common problem women reported was a colourless watery discharge (90.6%), followed by a black/brown discharge (33.3%) and foul‐smelling, pus‐coloured discharge (31.5%). Fewer women self‐reported bleeding (9.8%), severe abdominal pain (3.5%) and pain while urinating (0.6%) for an average of 3.3, 2.8 and 4.0 days, respectively (data not shown). Women reported that problems resolved in between 1 and 15 days with less than 5 days on average, and only 2.8% of women took pain medication. None sought help from a health provider or visited a heath facility. When asked if they would recommend this treatment, all women said they would, with most saying that it was quick and painless.

**Table 5:** Safety evaluation among participants who received thermal ablation treatment (*N* = 318)

	Number*	Per cent
Discomforts experienced during or immediately after thermal ablation		
Mild cramping	24	7.5
Moderate cramping	3	0.9
Nausea	6	1.9
Dizziness	16	5.0
Faintness/Vasovagal reaction	5	1.6
Flush/Hot	13	4.1
Bleeding/Spotting	32	10.0
Vaginal burns	1	0.3
Problems experienced in the month after treatment		
Colourless watery discharge	288	90.6
Brown/black discharge	106	33.3
Foul‐smelling, pus‐coloured discharge	100	31.5
Bleeding	31	9.8
Pain while urinating	2	0.6
Severe lower abdominal pain	11	3.5
Fever	0	0
What was done for problems		
Nothing	309	97.2
Took pain medication	9	2.8

* Participants could report more than one discomfort or problem experienced.

## Discussion

HPV and VIA screen‐positive women in Honduras who underwent thermal ablation treatment reported acceptability of the treatment, with low pain scores overall. These results are consistent with other studies that suggest that pain during thermal ablation is well tolerated and that most patients do not require analgesics for pain relief 9, 21, 22, 23. During the study period, across all government health facilities, there were no reported technical difficulties with the Liger device that prevented or delayed treatment, nor were there problems with the thermal ablation procedure.

There were few discomforts experienced during or immediately after the procedure. The patients’ self‐reported issues were minor, with the majority resolving within 3–4 days after the procedure, and none requiring additional medical attention. Findings from a recent meta‐analysis showed that women experienced only mild to moderate adverse effects after thermal ablation 23; our study supports these results.

There was no significant difference found in this study between the number of biopsies taken and the level of pain reported. However, based on the analysis of pain during the three pain‐assessment time periods, we speculate that a high pain level could be attributed to the biopsy procedure or pelvic examination, rather than solely the thermal ablation procedure. Once we ensured that the data collected on pain were specific to the thermal ablation procedure, we found that a greater proportion of women reported low pain scores. Given that thermal ablation is generally performed under a screen‐and‐treat approach without taking a biopsy, our findings could underestimate the level of acceptability in standard practice. In this study, we took biopsies on all participants prior to treatment, primarily for quality control and to evaluate treatment success at the one‐year follow‐up. We cannot conclude that there is no need for pain medication for the thermal ablation procedure, even in consideration of the confusion between pain from biopsy and pain from thermal ablation. During the first pain‐assessment time period, when medication was provided to most participants immediately after treatment, the majority of women reported having low pain levels even within a short time window for the drug to have an effect. It would be helpful for future studies to evaluate whether providing analgesics before or right after thermal ablation treatment is necessary in cases where biopsies will also be taken.

WHO recently issued its recommendation of thermal ablation for treatment of precancerous cervical lesions. While the question of pain associated with the procedure has been a major concern, several studies, including the present findings, have concluded that most women do not experience a high level of pain 23. This study was conducted within the same public health system where the current standard of care treatment is cryotherapy. In comparison with cryotherapy, thermal ablation requires a shorter length of treatment time 17 and does not require refrigerant gas. Evaluation of the thermal ablation procedure is ongoing, and the women in this study will be followed up at 12 months post‐treatment to evaluate the one‐year success rate of the procedure.

Our study has limitations. First, it is difficult to conclude with certainty whether the pain scores are reflective of the biopsy procedure, the thermal ablation procedure or a combination of both. In the third pain‐assessment time period when study staff emphasised that only pain experienced during the thermal ablation treatment should be reported, women may not have been able to distinguish between any pain experienced during either procedure. In addition, the three pain‐assessment time periods reported in this study were not intentional. They were determined to be necessary over the course of the study in order to clarify how pain was perceived and reported. Second, this study was not designed as a randomised clinical trial to compare pain experience between women undergoing thermal ablation and those receiving cryotherapy. Lastly, the four government health facilities included in the study were all located within the metropolitan region of Francisco Morazán. Consequently, the clients at these facilities may not be representative of the overall population in Honduras, and the findings may not be generalisable to the country as a whole, or to other low‐resource settings.

We used the Liger device in this study because it was the only handheld, battery‐powered thermal ablation device that was commercially available at the time of study design. Since that time, additional thermal ablation devices with similar characteristics became available. However, this study was not designed to compare devices.

In conclusion, thermal ablation was accepted and well tolerated by women undergoing treatment for precancerous cervical lesions. The procedure is safe based on post‐procedure observation at one month, and has minimal side effects, most of which diminish after a few days, with none requiring medical care. Based on our findings and those of similar studies, thermal ablation is a feasible and well‐accepted treatment option for standard care in low‐resource settings.
